# Micrognathia as a Diagnosis Marker for the Prenatal Identification of Edwards Syndrome

**DOI:** 10.3390/biomedicines13030573

**Published:** 2025-02-25

**Authors:** Cristina-Crenguţa Albu, Anca Daniela Brăila, Cristian-Viorel Poalelungi, Laurenţiu-Camil Bohîltea, Andreea-Mariana Bănățeanu, Constantin Marian Damian, Laurențiu Mihai Dîră, Claudia Florina Bogdan-Andreescu

**Affiliations:** 1 Department of Genetics, Faculty of Dentistry, “Carol Davila” University of Medicine and Pharmacy, 020021 Bucharest, Romania; cristina.albu@umfcd.ro; 2 Department of Obstetrics and Gynecology, University of Medicine and Pharmacy of Craiova, 200349 Craiova, Romania; anca.braila@umfcv.ro (A.D.B.); constantin.damian@umfcv.ro (C.M.D.); laurentiu.dira@umfcv.ro (L.M.D.); 3 Department of Obstetrics and Gynecology, Faculty of Medicine, “Carol Davila” University of Medicine and Pharmacy, 020021 Bucharest, Romania; 4 Department of Medical Genetics, Faculty of Medicine, “Carol Davila” University of Medicine and Pharmacy, 020021 Bucharest, Romania; 5 “Alessandrescu-Rusescu” National Institute for Maternal and Child Health, 20382 Bucharest, Romania; 6 Department of Speciality Disciplines, “Titu Maiorescu” University, 031593 Bucharest, Romania; andreea.banateanu@prof.utm.ro (A.-M.B.); claudia.andreescu@prof.utm.ro (C.F.B.-A.)

**Keywords:** congenital disorders, Edwards syndrome, prenatal screening, micrognathia, NIPT, karyotyping

## Abstract

**Background/Objectives:** Edwards syndrome, or trisomy 18, is a severe chromosomal disorder marked by numerous congenital anomalies, including micrognathia. This study evaluated the diagnostic significance of micrognathia as a prenatal indicator for trisomy 18 through a case series involving five confirmed instances. **Methods:** Ultrasound assessments concentrated on the inferior facial angle (IFA) and the jaw index, supplemented by Non-Invasive Prenatal Testing (NIPT) and karyotyping. **Results:** Micrognathia was consistently identified alongside other anomalies, reinforcing its reliability as an ultrasound marker for trisomy 18. **Conclusions:** The findings highlight the critical nature of early detection for informed parental counseling and effective pregnancy management.

## 1. Introduction

This study explores the diagnostic significance of micrognathia as a prenatal marker for trisomy 18, including its role in identifying both typical and mosaic forms of the condition. The nuanced presentation of mosaic trisomy 18 poses unique challenges in prenatal diagnostics, underscoring the need for detailed ultrasound examination and genetic testing. Congenital disorders, characterized by structural or functional anomalies arising during intrauterine life, pose a significant global health challenge [1]. These disorders are among the leading causes of morbidity and mortality in infants, mainly in high-income countries, where they account for approximately 30% of all deaths in children under the age of five [2]. 

The prevalence of congenital malformations and chromosomal disorders in Romania remains inadequately documented. Trisomy 21 (Down syndrome), the most common viable autosomal aneuploidy worldwide, is also the most frequently identified genetic disorder among newborns [3] and a leading cause of miscarriage [4]. Edwards syndrome (trisomy 18) and Patau syndrome (trisomy 13) follow in significance, with their incidence of genetic disorders in newborns estimated at 1.65% and 1.83%, respectively [3].

Edwards syndrome, or trisomy 18, is the second-most prevalent autosomal trisomy after Trisomy 21 (Down syndrome). It is associated with early-onset growth restriction and multisystem defects that can be identified through prenatal ultrasonography [5]. The condition arises from an extra chromosome 18, resulting in severe developmental abnormalities. Most affected fetuses do not survive to term, and those who are born face significant health challenges [6,7].

Additional genetic material disrupts normal development, leading to various physical and developmental anomalies. Timely detection of chromosomal abnormalities, such as Edwards syndrome, is essential for providing appropriate counseling and management options. Prenatal ultrasound is vital in detecting Edwards syndrome by identifying associated structural anomalies. Key ultrasound markers for this condition include increased nuchal translucency, growth restriction, and specific structural abnormalities such as micrognathia, heart defects, and clenched fists [8,9,10,11]. 

Micrognathia has been documented in studies of Edwards syndrome through fetal autopsy following termination of pregnancy or stillbirth [12,13,14], as well as in newborns [15,16,17,18,19,20,21]. In all reported cases, newborns exhibited respiratory distress due to micrognathia. This condition contributes to airway obstruction, leading to challenges in breathing, feeding, and the administration of general anesthesia when surgical intervention is required.

The prognosis for children with Edwards syndrome is typically poor, with most not surviving beyond infancy. Survival rates are disheartening: approximately 50% of infants with the condition do not make it past the first week of life, and only 5–10% survive until their first birthday [22,23]. Those who do survive face significant intellectual and physical disabilities [22,23]. 

In recent decades, the prevalence of trisomy 18 has increased due to the rising maternal age at conception [7]. The incidence of Edwards syndrome varies by country and is affected by local policies regarding pregnancy termination.

Early prenatal diagnosis plays a critical role in the management of Edwards syndrome. Micrognathia, an underdeveloped mandible, is particularly significant for early detection and is a reliable, non-invasive method for identifying at-risk fetuses during first-semester ultrasound. However, subtle or non-specific findings highlight the necessity of supplementing ultrasound with additional diagnostic tools, such as Non-Invasive Prenatal Testing (NIPT) and confirmatory invasive procedures like chorionic villus sampling (CVS) or amniocentesis. 

NIPT, which can be conducted as early as the 10th week of pregnancy, has established itself as a global standard for screening fetal aneuploidies [24]. The primary indications for NIPT include a known family history of genetic disorders or abnormal sonographic findings [25]. However, it is crucial to critically assess the limitations of NIPT. While this test offers high sensitivity and specificity for detecting conditions like trisomy 18, its reliability can be compromised by factors such as placental mosaicism. This condition can lead to discrepancies between the DNA profiles of the placenta and the fetus, potentially resulting in false positive or negative results. Moreover, the predictive value of NIPT is also influenced by the prevalence of the condition in the tested population. In populations with a low prevalence of trisomy 18, even a highly specific and sensitive test like NIPT can yield a relatively high rate of false positives, necessitating a careful interpretation of results. This underscores the critical need for comprehensive genetic counseling to ensure that patients understand the implications of NIPT results and the subsequent steps for confirmation. Such limitations highlight the necessity of confirmatory invasive testing following a high-risk NIPT result, emphasizing that NIPT should not be considered diagnostic but rather a preliminary screening tool. Consequently, positive NIPT results must be verified through invasive procedures such as CVS or amniocentesis, which provide direct insights into the fetal chromosomal status. These methods are critical for confirming the presence or absence of chromosomal abnormalities indicated by NIPT and ensuring accurate diagnosis and the appropriate management of the pregnancy. NIPT analyzes cell-free fetal DNA circulating in the maternal blood, originating from the placenta, and can detect trisomy 18 with high accuracy (over 99%) by evaluating the proportion of chromosome 18 present in the placenta-derived DNA [26]. Although it is highly sensitive and poses no risk to the mother or fetus, NIPT remains a screening test; thus, positive results necessitate confirmation through invasive testing [27,28]. These considerations are fundamental in prenatal care, where the accurate interpretation of screening results is crucial for the effective management of pregnancy and counseling. Ensuring that both patients and healthcare providers are aware of these nuances allows for informed decision-making and appropriate follow-up.

Ultrasound screening in the first trimester is typically conducted between 11 and 14 weeks of gestation and can identify physical markers that are indicative of trisomy 18. These markers include increased nuchal translucency and structural abnormalities such as micrognathia, cardiac defects, clenched fists, overlapping fingers, and growth restrictions [29,30,31,32]. Ultrasound is the primary imaging technique for detecting congenital anomalies due to its low cost, safety, ease of use, and widespread availability [33]. While it is non-invasive and provides valuable insights into fetal development, ultrasound may occasionally yield subtle or non-specific findings. Therefore, it is often combined with other tests to ensure a more comprehensive evaluation [34]. 

The main objective of prenatal ultrasonography is to reassure parents that fetal development is proceeding normally [35]. However, if an anomaly is discovered, parents need to be counseled about the nature and prognosis of the condition. Most congenital anomalies emerge early and can typically be detected between 10 and 16 weeks of pregnancy [36,37,38].

In addition to ultrasound, combined first-trimester screening (CFTS) can also be performed during the 11-to-14-week period. CFTS integrates an ultrasound measuring nuchal translucency with maternal blood tests that evaluate pregnancy-associated plasma protein A (PAPP-A) levels and human chorionic gonadotropin (hCG) [39]. An increase in nuchal translucency or abnormal maternal serum β-hCG levels may indicate a heightened risk for chromosomal abnormalities, including trisomy 18 [40]. These factors and maternal age generate an estimative risk for trisomy 18. While CFTS is a non-invasive test, it is essential to note that it provides only an estimation of risk rather than a definitive diagnosis.

Definitive diagnostic tests are recommended when a high risk for trisomy 18 is detected through NIPT, ultrasound, or CFTS. These tests include chorionic villus sampling (CVS), typically conducted between 10 and 14 weeks of gestation, and amniocentesis, usually performed after 15 weeks [41]. While CVS, typically conducted between 10 and 14 weeks of gestation, also samples placental tissue and may provide early genetic information, it may not accurately reflect the chromosomal status of the fetus, especially in cases of suspected mosaicism. Therefore, amniocentesis, usually performed after 15 weeks of gestation, is recommended to confirm NIPT results. Amniocentesis directly analyzes amniotic fluid containing fetal cells, providing a more definitive assessment of the fetal genome. CVS is a highly accurate technique for prenatal chromosomal analysis. Data collected from 48 laboratories involved in the European collaborative research on mosaicism in CVS (EUCROMIC) revealed a remarkable karyotyping success rate of 98.1%, with 62,865 out of 64,053 samples analyzed, resulting in a minimal failure rate of only 1.9% [42]. In the hands of experienced practitioners, it is safe and reliable, with a fetal loss risk that is comparable to amniocentesis [43]. Maternal cell contamination occurs in fewer than 1% of cases, particularly in inadequate sampling, which can lead to diagnostic errors [43]. In contrast, amniocentesis, which examines amniotic fluid, presents a slightly lower risk of miscarriage (less than 1%) [44,45]. CVS enables earlier diagnosis than amniocentesis, which can be advantageous for early pregnancy management. However, due to the early stage of pregnancy, CVS has some limitations, and amniocentesis remains the diagnostic test.

Early diagnosis is essential for assisting families and healthcare providers in making informed decisions regarding pregnancy management and preparing for potential challenges linked to trisomy 18. Micrognathia is a significant feature that may raise suspicion for trisomy and is often associated with other abnormal findings. 

This study emphasizes the diagnostic significance of ultrasound in identifying micrognathia as a marker for trisomy 18, focusing on several cases to illustrate its value in prenatal care. The primary objective of this study is to evaluate the diagnostic significance of micrognathia as a prenatal marker for Edwards syndrome, specifically within cases that were reassessed as second or third opinions. This retrospective analysis aims to demonstrate how micrognathia, identified during routine ultrasound examinations in the second trimester, contributes to the confirmation of chromosomal abnormalities associated with trisomy 18. By revisiting these cases, the study highlights the critical importance of micrognathia in prenatal diagnostics, emphasizing its potential to guide clinical decisions and improve outcomes through early and accurate detection.

Furthermore, while genetic factors are the principal cause of Edwards syndrome, emerging research indicates that environmental factors might also play a secondary role in the etiology of such chromosomal anomalies [6,7,46]. These factors encompass a variety of maternal health issues, such as nutritional deficiencies, exposure to environmental toxins, and uncontrolled maternal diseases [46]. In particular, the role of nutritional elements like folate is extensively studied; folate is vital for DNA synthesis and repair, particularly in the periconceptional period, to prevent neural tube defects and potential chromosomal abnormalities [47,48,49]. Exposure to environmental toxins such as heavy metals, pesticides, and industrial chemicals has also been scrutinized due to their teratogenic potential [50,51]. These agents can cause oxidative stress and DNA damage, which might contribute to nondisjunction events during meiosis, subsequently increasing the risk of aneuploidies [51,52]. Additionally, unmanaged maternal health conditions like diabetes and hypothyroidism can disrupt normal cellular processes and could predispose to chromosomal nondisjunction, suggesting the importance of comprehensive prenatal care that integrates environmental health management to mitigate risks associated with chromosomal abnormalities [53,54,55].

Understanding the interplay of these genetic and environmental factors is essential for advancing our knowledge of Edwards syndrome and enhancing the effectiveness of prenatal screening protocols. This study not only underscores the importance of micrognathia as a prenatal diagnostic tool but also advocates for an integrated approach in prenatal diagnostics and genetic counseling that considers both genetic predispositions and environmental influences.

## 2. Materials and Methods

This retrospective study analyzed a cohort of pregnancies examined at a private clinic in Bucharest, Romania, from 21 October 2020, to 31 March 2024. These cases were chosen based on previous ultrasound findings that had raised suspicions of genetic anomalies, specifically trisomy 18. The inclusion criteria focused on cases where micrognathia was observed during initial or subsequent ultrasound scans. These cases were then referred for further evaluation as a second or third opinion. Each case was re-evaluated to assess the presence and implications of micrognathia alongside other chromosomal abnormalities, utilizing both Non-Invasive Prenatal Testing and invasive procedures such as CVS or amniocentesis for confirmatory diagnoses. Data collection involved a comprehensive review of medical records, ultrasound reports, and genetic testing results, ensuring a thorough analysis of each case.

During this period, 3347 pregnant women were examined. Following specialized investigations, 30 pregnancies were prenatally diagnosed with various congenital fetal malformations, which raised suspicion of genetic anomalies. From this group, we isolated a study cohort comprising five pregnancies with micrognathia associated with another abnormal findings, which raised fears of chromosomal anomaly.

The standard protocol for these cases includes the following components:

(1) Advanced ultrasound imaging utilizes 3D/4D technology to offer comprehensive views of fetal anatomy.

(2) Sonographic examination is where the sonographer initially examines the fetal face through a subjective assessment aimed at detecting abnormalities, focusing on the presence of micrognathia. This evaluation enables an analysis of the midsagittal view of the fetal facial profile, including an assessment of the relationship between the mandible and the rest of the facial structure. Furthermore, this qualitative assessment is augmented by quantitative measurements of two key parameters: the inferior facial angle (IFA) and the jaw index [56]. The IFA is determined by drawing two intersecting lines in a midsagittal view of the fetal profile. The first line is established orthogonally to the vertical segment of the forehead, aligned at the level of the nasal bone synostosis. The second line extends from the tip of the chin to the anterior border of the most prominent point of the upper lip. The standard value for the IFA is approximately 65° (mean of 65.5° ± 8.13°) [57,58]. The jaw index is assessed using an axial view of the fetal mandible. Initially, a line is traced across the bases of the two rami, reflecting the laterolateral diameter. A subsequent line is drawn from the symphysis mentis to the midpoint of the laterolateral diameter, representing the anteroposterior diameter of the mandible. This jaw measurement is then normalized to the biparietal diameter (BPD), resulting in the jaw index [59]. The mean jaw index (along with its confidence interval) in prenatal ultrasound assessments is approximately 32.2 (32.2 ± 4.9) [59]. Values within this range are deemed normal, indicating a proportional relationship between the mandible and the skull. Deviations from this range may suggest abnormalities. Specifically, a jaw index below 24 indicates the potential for micrognathia (mandibular underdevelopment) [60], whereas a value below 21 has been linked to a 100% positive predictive value for diagnosing micrognathia [59]. 

(3) Non-Invasive Prenatal Testing (NIPT) is employed to detect potential chromosomal abnormalities.

(4) Genetic testing includes chorionic villus sampling (CVS) that is performed between 11 and 14 weeks of pregnancy, and amniocentesis that is conducted during the second trimester, between 15 and 20 weeks of gestation. Both procedures are used for fetal karyotyping to identify chromosomal abnormalities. In cases where NIPT indicates a high risk for trisomy 18, CVS is initially employed. It is important to recognize that while CVS provides valuable early genetic information through sampling placental tissue, it may not conclusively reflect the fetal chromosomal status in cases of suspected fetal mosaicism. Consequently, results from CVS alone are considered preliminary. Follow-up testing with amniocentesis is strongly recommended to provide a definitive diagnosis, as this method directly analyzes amniotic fluid containing fetal cells, thereby offering a more accurate representation of the fetal genomic status. Further investigations, including amniocentesis, are advised when CVS results indicate potential anomalies, ensuring the accuracy of initial findings and the management of complex cases identified through initial screenings.

(5) Parental Karyotyping is recommended in instances where an abnormal fetal karyotype is detected, to ascertain whether the chromosomal abnormality is inherited or has arisen de novo. This information is vital for genetic counseling and for planning future pregnancies.

The ultrasound examination was conducted transabdominally using a Voluson E10 ultrasound machine, BT18 (GE Healthcare, Wauwatosa, WI, USA), equipped with an RM6C three-dimensional/four-dimensional (3D/4D) volumetric probe. This advanced imaging technology allowed for the detailed visualization of fetal structures. 

Further investigations were recommended upon the identification of the micrognathia during the ultrasound scan. These included NIPT at the first step and CVS, or amniocentesis, to determine the fetal karyotype and check for any genetic abnormalities.

A parental karyotype from peripheral blood was also indicated if an abnormal fetal karyotype was detected. This additional step helps to identify whether any genetic anomalies are inherited or de novo, providing valuable information for genetic counseling and future pregnancy planning.

Medical staff provided patients with a clear explanation of the consent form detailing the clinical, paraclinical, and ultrasound examinations. The procedural steps necessary for conducting specialized investigations were also explained, along with the content of the consent form regarding the protection of personal data.

Ethics approval was obtained from the local Ethics Committee of the Alco San Medical Center in Bucharest, Romania. All parents signed the written informed consent form for participation in this study.

## 3. Results

The results are summarized in Table 1. The mean value for parental age is 32 for mothers and 37.2 for fathers. The mean gestational age is 25.8. The mean values for the IFA and the jaw index are 50.8 and 18.

Table 1 includes specific cases where CVS was initially used to confirm high-risk NIPT findings. It is important to highlight, as shown in cases 1 and 5, that while CVS indicated mosaic trisomy 18, this method samples placental tissue and may not fully reflect the fetal chromosomal status, particularly in instances of suspected mosaicism. Consequently, these results should be interpreted with caution. In case 5, where a 46,XX cell line was identified, further testing confirmed these were not maternal cells, indicating the necessity of robust confirmatory tests such as amniocentesis, which provides a definitive diagnosis by analyzing amniotic fluid containing fetal cells. This detailed approach underscores the complexity and the critical nature of comprehensive genetic testing in prenatal diagnostics.

In documenting the overall incidence and characteristic presentations of trisomy 18 within our cohort, we have identified critical associations between specific chromosomal patterns and phenotypic expressions of micrognathia. Our findings reveal a distinct correlation between homogeneous trisomy 18 and standard forms of micrognathia. More compelling, however, is the observed linkage between mosaic trisomy 18 and a moderate expression of micrognathia, quantified specifically with a jaw index of 19–20%. This consistent observation across our case subgroup not only reinforces the diagnostic value of micrognathia as a reliable sonographic marker for suspecting trisomy 18 but also enhances our understanding of its varied presentations depending on the underlying genetic landscape.

This granular insight into the nuanced relationships between sonographic markers and genetic variants sets a robust foundation for the subsequent discussion. It bridges our empirical data with the broader diagnostic and clinical narratives, inviting a deeper exploration of how these findings impact prenatal screening and genetic counseling methodologies. As we transition from these specific results to their broader implications, we aim to contextualize the potential of precise sonographic assessments in refining diagnostic protocols and improving patient outcomes.

**Figure 1:**
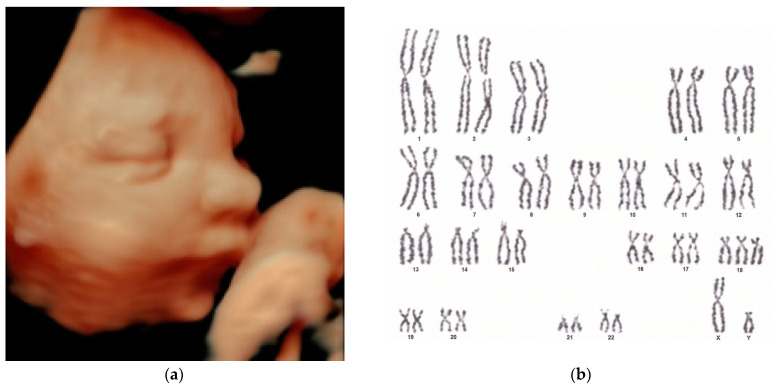
(**a**) Three-dimensional ultrasound evaluation of a fetal viscerocranium showing micrognathia, crucial for the initial clinical suspicion of chromosomal abnormalities; (**b**) fetal karyotype from amniotic cell culture 46,XY/47,XY,+18, confirming the presence of mosaic trisomy 18, which highlights the genetic complexity associated with the observed ultrasound features.

**Figure 2:**
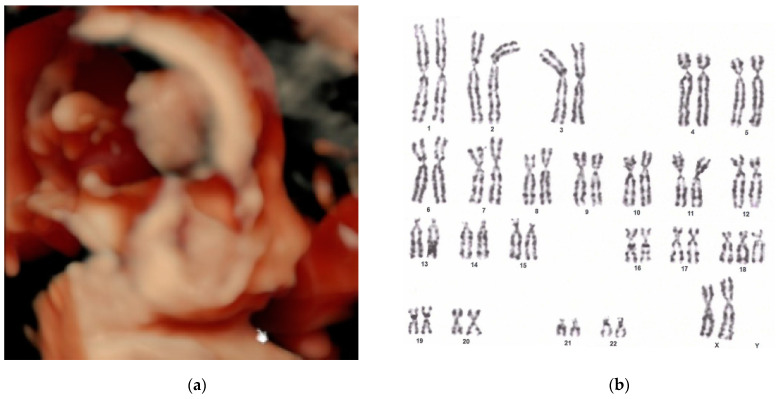
(**a**) Three-dimensional ultrasound of the fetal viscerocranium revealed retrognathia and micrognathia. These features significantly contributed to the heightened suspicion of a chromosomal disorder. (**b**) Fetal karyotype from amniotic cell culture 47,XX,+18. This result underscores the importance of comprehensive genetic analysis in prenatal diagnosis.

Figure 1 and Figure 2 illustrate representative images of cases from our study, each displaying features of micrognathia. These images are key in demonstrating the phenotypic markers that are critical for raising clinical suspicion of chromosomal disorders such as trisomy 18.

## 4. Discussion

Micrognathia is a prevalent craniofacial deformity, predominantly of congenital origin [56]. The etiology of this condition may be multifactorial, including hereditary transmission, random genetic mutations, or instances where no identifiable cause can be established. A retrospective study conducted in 2019 corroborated a genetic basis for micrognathia in 67% of the 41 fetuses examined [61]. Additionally, an investigation involving 18 cases of fetal micrognathia revealed that 28% were isolated anomalies, while 72% were correlated with other congenital defects [61]. Micrognathia is frequently associated with syndromic presentations encompassing a range of systemic anomalies and exhibits a strong genetic linkage [62]. Progress in genetic research has delineated various pathogenic genes implicated in syndromic micrognathia. This condition is often observed in conjunction with chromosomal disorders, including major trisomies, Turner syndrome, and the Pierre Robin sequence, as well as in multiple deletion and microdeletion syndromes such as DiGeorge syndrome and cri du chat syndrome [63,64,65]. 

The first case report of trisomy 18 was published by Edwards et al. in 1960 [66]. The characteristic facial phenotype associated with trisomy 18 encompasses micrognathia, a small triangular mouth, a short upper lip, a high palate, and a prominent occiput [67]. A study conducted in 1994, which analyzed 20 fetuses presenting with micrognathia and accompanying anomalies, discovered that 25% exhibited abnormal karyotypes, including five instances of trisomy, of which three were trisomy 18. Notably, 15% of these cases depicted micrognathia as the sole sonographic finding [68]. Facial anomalies are frequently identified in major trisomies. In a comprehensive study of 171 fetuses with confirmed trisomy 18, abnormal facial profiles were discerned in 41.5% [69]. A subsequent 2015 study that analyzed facial profile markers in 43 fetuses with trisomy 18 established nasal hypoplasia and micrognathia as prominent features [70]. Furthermore, a recent investigation into facial dysmorphisms in Edwards syndrome identified micrognathia in 62.5% of affected individuals [71]. 

Given the significant association between micrognathia and chromosomal abnormalities, identifying micrognathia during prenatal ultrasound necessitates careful evaluation and thorough searching for possible associated abnormalities. It is noteworthy that in the cases reviewed, the mothers were reported to be healthy and young, which underscores the genetic roots of the condition rather than environmental or maternal health factors contributing significantly to these cases.

The identification of micrognathia through advanced ultrasound serves as both a diagnostic and prognostic marker for Edwards syndrome. Its strong association with homogeneous and mosaic trisomy 18 enhances early diagnostic and targeted genetic counseling, reinforcing the critical link between phenotypic characterization and chromosomal abnormalities. Moreover, the differentiation between moderate and more severe forms of micrognathia, correlating distinctly with mosaic patterns of trisomy 18, highlights an important area for clinical attention.

Our research supports the integration of systematic sonographic screening with confirmatory genetic testing to improve the accuracy of trisomy 18 detection. Finally, micrognathia arises as a particular, non-invasive marker, reinforcing its role in early, precise, personalized prenatal care.

This study underscores the critical role of micrognathia in the prenatal diagnosis of Edwards syndrome, illustrating its potential as a non-invasive marker that is detectable through advanced ultrasound techniques. The correlation between the ultrasound findings and genetic testing results highlights the effectiveness of this approach in early prenatal screenings. Such findings not only affirm the diagnostic value of micrognathia but also emphasize the broader applicability of sonographic markers in the early detection of genetic disorders.

In response to potential queries regarding the sample size of this study, it is important to highlight the specific focus and detailed examination of each case, which is justified given the rare and complex nature of Edwards syndrome. Although the number of cases in this study is limited, each instance is meticulously documented and analyzed, providing a depth of insight that is both clinically relevant and scientifically valuable. The rarity of the condition and the diagnostic specificity required for its prenatal identification mean that even a single, well-documented case can significantly advance our understanding and improve diagnostic accuracy. Therefore, this study, through its concentrated examination of micrognathia as a diagnostic marker for Edwards syndrome, contributes important knowledge to the field, enhancing the capabilities of prenatal screening and the precision of genetic counseling.

In this study, five cases were evaluated as second and even third opinions, with ultrasound examinations conducted during the second trimester of pregnancy. All five cases exhibited micrognathia, which contributed to the diagnosis of chromosomal anomalies alongside other observed abnormalities. 

Micrognathia is characterized by the developmental hypoplasia of the mandible, resulting in a small jaw size and an overbite in profile view [62,72]. This condition is one of the most common craniofacial deformities and is often associated with retrognathia, glossoptosis, and upper-airway obstruction. These complications can significantly affect infants’ appearance, complicate feeding, and pose serious risks to their survival [73].

Micrognathia can occur sporadically or as part of an inherited condition and may be isolated or manifest within a broader syndrome [74]. 

Mild cases of micrognathia may be considered a normal variant and often go undetected during routine scans. However, more prominent instances are frequently linked to a variety of chromosomal and genetic syndromes, including Pierre Robin sequence, Treacher Collins syndrome, branchio-oculofacial syndrome, cri du chat syndrome, as well as trisomy 13, 18, and 9 [60,68,75,76,77].

Detecting micrognathia during prenatal assessments has implications for prenatal and postnatal management, primarily due to its strong association with various abnormalities and syndromes. Measurements of the inferior facial angle (IFA) play an important role in evaluating fetal facial contours and allow the early identification of anomalies during pregnancy. 

In this research, all micrognathia instances were isolated, sporadic, syndromic, and non-hereditary. Notably, two of these cases involved women experiencing their second pregnancy, with no reported genetic anomalies in their first pregnancy. This pattern suggests that micrognathia and its associated abnormalities may not arise from genetic inheritance, raising the possibility of teratogenic factors as a potential cause. 

Genetic anomalies can result from various factors influencing the structure or number of chromosomes or altering DNA sequences. These factors may be inherited or acquired over an individual’s lifetime. The primary contributors to genetic anomalies include spontaneous mutations (errors in DNA replication), errors during meiosis, inherited genetic mutations, environmental factors (mutagens) such as radiation, tobacco smoke, pesticides, industrial chemicals, viruses, advanced parental age (particularly maternal age), epigenetic changes, mitochondrial DNA mutations inherited from the mother, and copy number variations [78,79,80,81].

In this study, we could not establish a link between advanced paternal age and Edwards syndrome due to the limited sample size and the average parental ages falling within typical reproductive ranges: 32 years for mothers and 37.2 years for fathers. Advanced maternal age is generally considered to be over 35 years of age for women [82,83]. In contrast, advanced paternal age is typically defined as over 40 years of age for men, associated with a substantial decline in semen quality [84,85]. Therefore, given that the average paternal age in our study is below this threshold, the findings do not indicate a significant effect of paternal age on the occurrence of Edwards syndrome. 

Moreover, no specific mutagenic environmental factors—such as radiation, pesticides, or industrial chemicals—were identified in these cases. The absence of documented exposure to known risk factors limits our ability to link Edwards syndrome to environmental mutagens. Consequently, the origin of Edwards syndrome in this series remains unclear, with no influences from environmental factors or paternal age identified. 

To further explore the connection between micrognathia and the other observed abnormalities, NIPT was recommended. NIPT offers a reliable, non-invasive method for detecting chromosomal anomalies associated with conditions such as Edwards syndrome. By analyzing fetal DNA circulating in maternal blood, NIPT can help clarify whether micrognathia and the additional abnormalities point to a chromosomal disorder, aiding in more accurate prenatal diagnosis and management planning.

NIPT can be effectively utilized to diagnose trisomy 18, but to differentiate between false and true positives, associated anomalies such as cardiac defects, craniofacial abnormalities, and limb malformations must be searched. The association between micrognathia, additional anomalies, and positive NIPT is essential for confirmation. Multiple structural anomalies associated with micrognathia significantly increase the likelihood of an NIPT true-positive result, reinforcing the need for fetal karyotyping to ensure precise diagnosis.

Among these associated anomalies, trisomy 18 may lead to asymmetrical growth restriction, where the trunk is more profoundly affected than the head. This asymmetry can be identified using 3D ultrasound technology and VOCAL techniques to measure embryo volume, serving as an early indication of growth restriction in high-risk pregnancies [86,87].

In this study, four out of five expectant mothers underwent NIPT, with all results indicating trisomy 18. This strong correlation between NIPT findings and micrognathia and other abnormalities highlights the value of NIPT as a diagnostic tool for identifying Edwards syndrome in pregnancies characterized by craniofacial and other structural anomalies. These results strengthen the association between trisomy 18 and specific prenatal ultrasound markers, allowing early and accurate diagnosis. 

In all instances, karyotyping was conducted to confirm the diagnosis of aneuploidy. Two cases involved chorionic villus sampling (CVS), while the other three were confirmed through amniocentesis. Karyotype results validated the presence of trisomy 18 in each case, corroborating the findings from NIPT and definitively diagnosing Edwards syndrome. 

While NIPT is entirely safe for both the mother and fetus, functioning as a non-invasive screening tool, it does require confirmation via karyotyping for a definitive diagnosis. In this study, trisomy 18 was confirmed in all cases identified through NIPT. This result contrasts with a recent survey by Gug et al. [88], suggesting that trisomy 21 was the only chromosomal anomaly consistently confirmed through NIPT in all high-risk cases. These findings indicate that, besides trisomy 21, NIPT can be highly accurate in detecting trisomy 18, supporting its broader reliability as an early screening method for multiple chromosomal abnormalities.

In our study, the integration of ultrasound findings and NIPT demonstrated a high predictive accuracy for trisomy 18, reflecting a strong concordance between these diagnostic modalities in single pregnancies. The recent literature has indicated that false-negative results in aneuploidy testing may arise from cases involving a vanishing or aborting twin, which can compromise the accuracy of the test [89,90,91]. Conversely, false-positive results are frequently associated with confined placental mosaicism [92]. The incidence of false-negative results has fluctuated, with a report of 7.3% in a 2016 study [93] and a lower rate of 2.07% in a more recent study from 2022 [94]. 

The sensitivity of NIPT for detecting trisomy 18 stands at 98.2% for the Panorama test and 97.4% for the Harmony test [95]. While NIPT is an effective screening tool, its accuracy is significantly improved when corroborated by ultrasound findings. In our study, an IFA of less than 65˚ and a jaw index of less than 20%, alongside additional structural anomalies, increased the suspicion of chromosomal abnormalities. 

Additionally, it is noteworthy that the prenatal diagnosis of micrognathia can be made as early as the first trimester [60], facilitating early attention to other structural anomalies and the potential diagnosis of genetic disorders, such as trisomy 18. This capability for early detection has significant implications for clinical management, providing families with timely counseling and options. 

Managing a pregnancy diagnosed with trisomy 18 necessitates a multidisciplinary approach that is adapted to the family’s needs as well as the medical status of both the mother and fetus. This encompasses comprehensive prenatal counseling, medical monitoring, and individualized care planning to address both medical and emotional aspects [96].

Genetic specialists provide essential information to parents regarding the prognosis of trisomy 18, which typically includes severe intellectual disabilities, congenital heart defects, and a high risk of stillbirth or early neonatal death. Embryonic chromosomal abnormalities, including trisomies, are well-documented causes of early spontaneous abortions [97]. Families receive support in making informed choices about whether to continue or terminate the pregnancy, with thorough counseling and discussion of potential outcomes [98]. For parents who decide to continue, additional care planning is coordinated to ensure appropriate support and resources are available throughout the pregnancy and beyond. 

In this study, four out of five couples chose to terminate the pregnancy after receiving a diagnosis of trisomy 18. Notably, three of these cases opted for abortion despite being late in the second trimester or even in the third trimester. The primary reasons for their late decision included the desire for updated information regarding fetal health issues, prompting them to seek second and even third opinions. 

This fact reflects the complexity of decision-making in the context of a severe diagnosis. It highlights the importance of providing comprehensive support and information to families facing such challenging circumstances. The findings from our study not only augment the existing literature on the prenatal diagnosis of trisomy 18 but also pave the way for advancements in how such conditions are approached and managed clinically. Our research underscores the necessity for ongoing innovations in prenatal screening technologies and methodologies, ensuring that expectant families receive the most accurate and actionable health information possible. Future research should aim to expand on these findings, exploring the full spectrum of genetic anomalies associated with micrognathia and other sonographically detectable markers to further refine and personalize prenatal diagnostic processes.

Moreover, our findings highlight a distinct correlation between the fetal karyotype and the phenotypic aspects of micrognathia. This nuanced understanding of micrognathia’s presentations, aligned with the underlying genetic landscape, not only reinforces its diagnostic value but also enhances the precision of our prenatal assessments. These detailed insights into the relationships between sonographic markers and genetic variants emphasize the importance of advanced diagnostic protocols that incorporate both phenotypic and genotypic data. 

Additionally, this study enriches the diagnostic landscape by illustrating the potential of micrognathia not just as a marker, but as a critical component in the constellation of prenatal assessment tools that contribute to the comprehensive evaluation of fetal health. It emphasizes the role of detailed phenotypic assessment in enhancing the predictive accuracy of genetic screenings.

Micrognathia does not diagnose chromosomal anomalies by itself. However, finding micrognathia should prompt a thorough investigation for related anomalies. In the case of micrognathia and associated anomalies, NIPT is recommended.

Our findings advocate for a multidisciplinary approach to prenatal diagnosis, combining sonographic evaluation with genetic insights to offer a more nuanced understanding of fetal health. This approach not only aids in early detection and intervention strategies but also supports informed decision-making by healthcare professionals and expectant families.

## 5. Suggestions for Future Research 

Future research should focus on validating these findings through more extensive, multi-center studies to improve the generalizability and reliability of the association between micrognathia and trisomy 18. Prospective studies could allow for standardized data collection and consistent measurement techniques, enhancing the diagnostic utility of the inferior facial angle (IFA) and the jaw index. Exploring genetic predispositions and environmental factors associated with micrognathia and trisomy 18 could provide valuable prenatal counseling and risk assessment insights. Additionally, a longitudinal study following affected cases postnatally could offer essential information on developmental outcomes, aiding in prognosis and management strategies for fetuses with prenatal markers of trisomy 18.

## 6. Limitation of the Study

This study is limited by its small sample size of five cases, which restricts the statistical power and generalizability of the findings. A larger cohort would enable more robust conclusions regarding the association between micrognathia and trisomy 18. Additionally, the retrospective design confines the analysis to available records, which may vary in detail and consistency. As a single-center study, the findings may not fully represent broader populations, as regional and demographic factors can influence prenatal conditions. Furthermore, focusing on cases referred for a second opinion introduces potential selection bias, likely over-representing severe or ambiguous cases and potentially skewing the data towards more complex presentations of trisomy 18.

## 7. Conclusions

Early prenatal diagnosis of Edwards syndrome is essential for informed medical decision-making and the effective management of the pregnancy. 

Micrognathia, a common craniofacial abnormality associated with trisomy 18, is a valuable diagnostic marker in prenatal ultrasound screenings. Further genetic testing is necessary to confirm the diagnosis in cases where micrognathia is detected alongside other anomalies. Our study showed that trisomy 18 was confirmed in all cases initially identified through NIPT, which underscores the reliability of this screening tool for identifying this chromosomal disorder.

## Figures and Tables

**Table 1:** The summative table with subjects and data analyses.

Mother Age	Father Age	Obstetric History	Gestational Age (Weeks)	Ultrasound Findings	Oro-Facial Modifications	IFA	Jaw Index (%)	NIPT Result	Fetal Karyotype	Observations
41	50	Second pregnancy	13	Male monofetal pregnancy, hemi ventriculomegaly, lissencephaly	Unilateral cleft lip and palate, retrognathia, micrognathia	55	20	High risk for trisomy 18	CVS at 13 weeks: *mosaic trisomy 18* 46,XY/47,XY,+18 *	Termination of pregnancy
25	29	First pregnancy	19	Male monofetal pregnancy, polyhydramnios, esofagean atresia	Micrognathia, one deglutition at 18 min	50	18	Intermediate risk for trisomy 18	Amniocentesis at 19 weeks: *trisomy 18*; 47,XY,+18	Continuation of pregnancy
24	28	First pregnancy	20	Female monofetal pregnancy, ventriculomegaly, lissencephaly, dolichocephaly	Micrognathia	53	19	High risk for trisomy 18	Amniocentesis at 20 weeks: *mosaic trisomy 18* 46,XX/47,XX,+18	Termination of pregnancy, smoker
31	35	Second pregnancy	21	Female monofetal pregnancy, ventriculomegaly, hydrocephalus, corpus callosum hypoplasia, choroid plexus cyst, diaphragmatic hernia, intrauterine growth restriction	Retrognathia, micrognathia	47	17	No	Amniocentesis at 21 weeks: *trisomy 18* 47,XX,+18	Termination of pregnancy, smoker
39	44	First pregnancy	14	Female monofetal pregnancy, ventriculomegaly, lissencephaly	Retrognathia, micrognathia	49	16	High risk for trisomy 18	CVS at 14 weeks: *mosaic trisomy 18*; 46,XX/47,XX,+18 **	Termination of pregnancy

* CVS results may not conclusively reflect the fetal chromosomal status due to potential placental mosaicism. Follow-up with amniocentesis is recommended for definitive diagnosis. ** Additional molecular tests confirmed that the 46,XX cell line was fetal, not maternal contamination.

## Data Availability

The data presented in this study are available on request from the corresponding author.
